# ASSOCIATIONS BETWEEN UNMET NEED AND SELF-REPORTED HEALTH STATUS AND QUALITY OF LIFE IN CHINESE OLDER ADULTS

**DOI:** 10.1093/geroni/igae098.3393

**Published:** 2024-12-31

**Authors:** Siou-Tang Huang, Weiguo Chong, Herng-Chia Chiu

**Affiliations:** China Medical University, Taichung, Taichung, Taiwan (Republic of China); Tsinghua University, Beijing, Beijing, China (People’s Republic); Tsinghua University, Beijing, Beijing, China (People’s Republic)

## Abstract

**Background:**

The demand for home-based community health services has become more critical as the population ages. To what extent are associations between unmet need and health status crucial for health planning for older adults. The study aimed to examine the degree of unmet need and self-reported health status and quality of life for an older adult population.

**Methods:**

Population-based data from the China Health and Longevity Longitudinal Survey (CLHLS) was used. Older persons who participated in both the 2014 and 2018 surveys were included. After match and exclusion, 2,271 subjects were analyzed. Community health services include services of home visits, mental and health prevention.

**Results:**

The baseline age is 80.3 years old, 84.17 years old on average four years after. More than 10% of older persons lost their spouses during this period. There are 46.40% and 46.63% reported unmet need in 2014 and 2018, respectively. LMM analysis reveals that compared with the unmet group, those reported needs were fully met and partially met older appeared significantly better in health status (ß = 0.12, ß =0.01) and quality of life (ß =015, ß =0.11).

**Implication:**

Our findings conclude that unmet services are highly negatively associated with health status as well as quality of life. To improve the older population’s health and quality of life, a community services survey is needed to design programs to meet the needs of various older populations.

